# [^177^Lu]Lu-PSMA radioligand therapy in systemic therapy-naïve metastatic hormone-sensitive prostate cancer patients: a retrospective study

**DOI:** 10.1007/s00259-026-07802-9

**Published:** 2026-02-13

**Authors:** Ilva K. Langrate, Heidemarie Ofner, Elisabeth Kretschmer-Chott, Holger Einspieler, Oana C. Kulterer, Stefan Schmitl, Lukas Nics, Shahrokh F. Shariat, Marcus Hacker, Sazan Rasul, Gero Kramer

**Affiliations:** 1 Department of Biomedical Imaging and Image-Guided Therapy, Division of Nuclear Medicine, Medical University of Vienna, Währinger Gürtel 18-20, Vienna, 1090 Austria; 2 Department of Urology, Comprehensive Cancer Center, Medical University of Vienna, Vienna, Austria; 3 Department of Biomedical Imaging and Image-Guided Therapy, Division of General and Pediatric Radiology, Medical University of Vienna, Vienna, Austria; 4 Department of Urology, University of Texas Southwestern Medical Center, Dallas, USA; 5 Division of Urology, Department of Special Surgery, The University of Jordan, Amman, Jordan; 6 Department of Urology, Second Faculty of Medicine, Charles University, Prague, Czech Republic; 7 Department of Urology, Weill Cornell Medical College, New York, USA; 8 Karl Landsteiner Institute of Urology and Andrology, Vienna, Austria

**Keywords:** Prostate cancer, [^177^Lu]Lu-PSMA-RLT, MHSPC, Hormone-naïve, Radioligand therapy

## Abstract

**Purpose:**

Lutetium-177-labelled radioligand therapy targeting prostate-specific membrane antigen ([¹⁷⁷Lu]Lu-PSMA-RLT) is an established third-line treatment for metastatic castration-resistant prostate cancer (mCRPC). We evaluated the therapeutic effectiveness of [¹⁷⁷Lu]Lu-PSMA-RLT in systemic therapy-naïve patients with metastatic hormone-sensitive prostate cancer (mHSPC).

**Patients and methods:**

This retrospective analysis included mHSPC patients without prior systemic hormone therapy or chemotherapy who received [¹⁷⁷Lu]Lu-PSMA-RLT between September 2015 and February 2025. The primary outcomes were prostate-specific antigen (PSA) response one month after the final cycle and PSA progression-free survival (PFS). Hematological and biochemical laboratory parameters were monitored to assess toxicity. Baseline PSMA PET imaging parameters (maximum standardized uptake value (SUVmax), mean SUV, peak SUV, and total tumor volume (TTV)) were collected. Uni- and multivariable analyses, together with Kaplan-Meier survival analysis, were performed to identify predictors of PSA-PFS, androgen deprivation therapy (ADT)-free survival, and any-therapy-free survival.

**Results:**

Twenty-four patients were identified. Median PSA declined significantly from 6.4 ng/mL (range 0.49–3149) at baseline to 2.3 ng/mL (range 0.00-1731) one month after last [¹⁷⁷Lu]Lu-PSMA-RLT cycle (*p* = 0.002). All patients received ≥ 3 cycles of [^177^Lu]Lu-PSMA-RLT. Overall, 15 of 24 patients (62.5%) achieved a PSA decline ≥ 50%, including 10 patients (41.7%) with a decline ≥ 80%. Median PSA-PFS was 24.6 months in patients with a PSA decline ≥ 50%, versus 5.2 months in those with a decline < 50% (*p* < 0.0001). In the Kaplan-Meier analysis, baseline TTV, the presence of bone metastases, and age < 66.3 years were significant predictors of shorter ADT-free and any-therapy-free survival (all *p* < 0.05). In contrast, baseline SUV-values of metastatic lesions showed no predictive value in distinguishing responders from non-responders. No severe treatment-related adverse events occured. One patient (4.2%) died 10.3 months after initiation of [¹⁷⁷Lu]Lu-PSMA-RLT.

**Conclusion:**

This study evaluated [¹⁷⁷Lu]Lu-PSMA-RLT in early-stage PCa, demonstrating strong PSA decline, prolonged PCa progression-free period, and good tolerability. Baseline TTV and metastatic extent were independent predictors of ADT-free and any-therapy-free survival. The absence of differences in PSMA PET-derived SUV-parameters between responders and non-responders suggests a more homogeneous tumor biology in treatment-naïve mHSPC patients. Prospective studies are needed to define the clinical benefit and predictors of response to [¹⁷⁷Lu]Lu-PSMA-RLT in this setting.

## Introduction

In 2020, prostate cancer (PCa) accounted for 23.2% of male cancer cases in the European Union, with nearly half occurring in men aged 45–69 [1]. While PCa primarily affects older men, incidence in younger populations is rising worldwide [2]. Systemic PCa treatments, including androgen-deprivation therapy (ADT) or chemotherapy, often cause long-term complications highlighting the need for therapy options that prolong progression-free survival (PFS) while preserving quality of life (QoL) [3].

Prostate-specific membrane antigen (PSMA) is highly expressed in malignant PCa cells but minimally in benign tissues [4], making it an attractive target for disease imaging and therapeutic interventions. New imaging modalities, using PSMA-targeting radiotracers such as ^68^Ga-PSMA, have demonstrated higher sensitivity and specificity for PCa detection, prognostics and treatment monitoring compared with conventional imaging (computational tomography, magnetic resonance imaging) and tumor markers (serum PSA) [5, 6]. Radioligand therapy (RLT) utilizes PSMA-binding ligands or antibodies labeled with the radioactive isotope [^177^Lu]lutetium [7] delivering targeted cytotoxic β^−^ radiation to PCa cells [8]. Since March 2022, the European Association of Urology (EAU) recommends [¹⁷⁷Lu]Lu-PSMA-RLT as a third-line therapy for PSMA-positive metastatic castration-resistant prostate cancer (mCRPC) patients who have progressed after one taxane chemotherapy and one androgen receptor pathway inhibitor (ARPI) therapy, based on the encouraging results of the VISION trial [9]. Since March 2025, the Food and Drug Administration (FDA) has approved lutetium Lu-177 vipivotide tetraxetan (Pluvicto, Novartis Pharmaceuticals Corporation) for mCRPC patients in the taxane-chemotherapy-naïve setting, following the promising results of the PSMAfore trial [10].

Only a few studies, including UpfrontPSMA and Lutectomy [11, 12], have investigated the therapeutic effects of [¹⁷⁷Lu]Lu-PSMA-RLT in metastatic hormone-sensitive prostate cancer (mHSPC). These studies demonstrated improved efficacy and safety profile, supporting earlier use of [¹⁷⁷Lu]Lu-PSMA-RLT. The ongoing international phase III PSMAddition trial (NCT04720157) is evaluating [^177^Lu]Lu-PSMA-617 plus standard-of-care (ADT and ARPI) versus standard-of-care (SOC) alone in treatment- naïve or minimally-treated men with PSMA-positive mHSPC [13]. After randomization (1:1) patients receive up to six cycles of 7.4 GBq [¹⁷⁷Lu]Lu-PSMA-RLT every six weeks in addition to SOC, or SOC alone. Radiographic progression-free survival is the primary endpoint, with overall survival (OS), PSA response, time to CRPC, safety and quality of life as secondary endpoints [14]. The anticipated results could prove greater benefit of [^177^Lu]Lu-PSMA-RLT over currently approved treatment options in the mHSPC setting, given that patients in this disease stage have better preserved organ functions and less advanced tumor biology compared to mCRPC [15].

In this study, we evaluated systemic therapy-naïve mHSPC patients treated with [^177^Lu]Lu-PSMA-RLT. We assessed the therapeutic effects of [^177^Lu]Lu-PSMA-RLT, focusing on PSA response rate, PSA progression-free survival (PSA-PFS), toxicity, and time to subsequent systemic therapies.

## Patients and methods

### Patients

In this retrospective analysis, we studied all PCa patients treated with [^177^Lu]Lu-PSMA-RLT at the Department of Nuclear Medicine, Vienna General Hospital, between September 2015 and February 2025. We aimed to identify mHSPC patients who had not received any prior systemic therapy for prostate cancer with a follow-up period of at least 6 months after the last therapy cycle. All cases were evaluated by a multidisciplinary oncology board, which approved [¹⁷⁷Lu]Lu-PSMA-RLT as the initial treatment with written informed consent obtained from all patients prior to therapy. Demographic, treatment history, diagnostic, and follow-up laboratory data were retrieved from the Vienna General Hospital Information Management System.

### PET imaging and image analysis

The prerequisite for receiving [^177^Lu]Lu-PSMA-RLT was the presence of PSMA-positive lesions on a [^68^Ga]Ga-PSMA-11 whole-body PET scan, performed for all patients prior to initiation of treatment. The [^68^Ga]Ga-PSMA-11 PET protocol consisted of intravenous administration of 2 MBq/kg body weight of [^68^Ga]Ga-PSMA-11, administered 60 min before image acquisition. Scans were obtained using either PET/CT (Biograph TruePoint 64; Siemens, Erlangen, Germany), PET/CT (Biograph 128 Vision Quadra Edge; Siemens, Erlangen, Germany), or PET/MRI (3.0-Tesla Biograph MMR system; Siemens, Erlangen, Germany). Additionally, a second [^68^Ga]Ga-PSMA-11 whole-body PET scan was performed for all patients to visually assess therapy response 4–6 weeks after the last cycle of [^177^Lu]Lu-PSMA-RLT. Image analysis was conducted using Hybrid 3D software (version 4.17, Hermes Medical Solutions, Stockholm, Sweden). PET image intensities (Bq/mL) were converted into standardized uptake values (SUV) normalized to body weight. Cuboidal volumes of interest (VOIs) with target size of 3 mL were manually placed in the right liver lobe to determine liver SUVmean as a reference threshold. PSMA-positive lesions were defined as foci with SUVmax exceeding the liver SUVmean; lesions were then segmented, and SUV parameters, including TTV, were collected.

### Therapy cycle overview

From September 2015 until 2021, patients received [^177^Lu]Lu-PSMA-617, prepared according to in-house manufacturing protocols, every 4 weeks at a median dose of 7.45 ± 0.43 GBq per cycle. Since 2021, patients have been treated with [^177^Lu]Lu-PSMA-I&T, produced and quality controlled according to in-house protocols and administred every 6 weeks, receiving a median dose of 7.44 ± 0.5 GBq per cycle. The therapy was administered in accordance with § 8 of the Austrian Medicines Act.

During each therapy cycle, patients received antiemetic medication (Ondansetron) and 1000 ml of 0.9% NaCl for nephroprotective hydration 30 min before and after [^177^Lu]Lu-PSMA-RLT administration. Patients were advised to increase fluid intake to reduce risk of nephrotoxicity. At each admission, patients underwent a physical examination by an experienced nuclear medicine physician. Performance status was evaluated using the Karnofsky Index and the Eastern Cooperative Oncology Group (ECOG) scale. Hematological (hemoglobin, leukocytes, thrombocytes) and biochemical (creatinine, alkaline phosphatase (AP), lactate dehydrogenase, testosterone, PSA) parameters were collected before, during, and one month after the last RLT cycle. Estimated glomerular filtration rate (eGFR) was calculated using Chronic Kidney Disease Epidemiology Collaboration formula for male [16]. Therapy toxicity was evaluated using the Common Terminology for Adverse Events (CTCAE) version 5.0 [17].

### Statistical analysis and clinical outcome calculations

Statistical analyses were conducted using SPSS 28.0 (IBM, Armonk, NY, USA) and data visualization was performed using RStudio^®^ (version 2024.12.1 + 563; Posit Software, PBC, Boston, MA). Data distributions were assessed with the Kolmogorov-Smirnov test. Normally distributed variables are shown as mean and standard deviation (SD), while not-normally distributed variables are presented as median and range, and were log₁₀- transformed for analysis. Categorical variables are presented as counts and percentages. The median PSA-PFS, ADT-free survival and any-therapy-free survival corresponding 95% confidence intervals (CI) for each patient group and overall cohort were calculated using Kaplan-Meier analysis.

PSA response was defined as the percentage change from baseline to post-therapy value, with a ≥ 50% decline regarded as clinically significant. Patients with a PSA decrease ≥ 50% after RLT were classified as “responders”, whereas those with a decline < 50% or without any PSA decline were classified as “non-responders”. PSA-PFS was defined according to the Prostate Cancer Clinical Trial Working Group 3 (PCWG3) criteria as the time from treatment initiation to PSA progression (> 25% and ≥ 2 ng/ml above nadir) [18] or last follow-up, if not reached. Any-therapy-free survival was defined as the period between the first RLT cycle to the initiation of any next therapy (chemotherapy, hormone therapy, radiotherapy, further radioligand therapy), or last follow-up for patients who had no further treatment. ADT-free survival was defined as interval from the first RLT cycle to initiation of ADT or last follow-up, if ADT was not started. Time periods were recorded in days and converted to months by dividing the number of days by 30.4. Predictors of PSA response (PSA decline ≤ 50% versus > 50%) in relation to baseline clinical (body mass index (BMI), previous radical prostatectomy, metastasis location), treatment-related (cycle count) and PSMA PET parameters (TTV, SUVmax, SUVmean, SUVpeak) were assessed using univariable binary logistic regression models. Kaplan-Meier analysis with log-rank tests and univariable Cox regression were used to explore associations between baseline factors and PSA-PFS, ADT-free survival, and any-therapy-free survival. Implied variables included baseline clinical, laboratory and imaging parameters: age at the start of RLT, Gleason score, number of RLT cycles, BMI, metastasis location, primary therapy, laboratory parameters including baseline hemoglobin, leukocytes, thrombocytes, creatinine, lactate dehydrogenase, alkaline phosphatase, PSA and post-therapy PSA value and imaging parameters (TTV, SUVmax, SUVmean, SUVpeak). Parameters reaching significance (*p* < 0.05) on univariable analysis were then included for multivariable Cox regression.

## Results

A total of 533 patients who received at least one cycle of [¹⁷⁷Lu]Lu-PSMA-RLT were studied. Of these, only 24 mHSPC patients with no history of prior systemic PCa treatment were identified. The mean age at the time of the first RLT cycle was 66.3 ± 5.4 years. The majority of these patients were in good physical condition, with an ECOG performance status of 0 (95.8%) or 1 (4.2%). All patients had a Karnofsky performance score above 80% (24/24 patients). Included patients received between 3 and 6 cycles of [^177^Lu]Lu^−PSMA^-^RLT^. Nine patients (37.5%) received 3 RLT cycles; 2 patients (8.3%) 4 RLT cycles; 4 patients (16.7%) 5 cycles, and 9 patients (37.5%) 6 cycles of [^177^Lu]Lu^−PSMA^-^RLT^. Additional clinical characteristics are shown in Table 1.

**Table 1:** Demographic characteristics of systemic therapy-naive mHSPC patients prior to [^177^Lu]Lu-PSMA-RLT

Patients (*n*)	24
Age (mean ± SD)	66.3 ± 5.4
BMI (mean ± SD)	27.5 ± 4.1
ECOG performance score (n (%))	
0	23 (95.8%)
1	1 (4.2%)
Karnofsky score (n (%))	
≥ 80%	24 (100%)
Comorbidities (n (%))	
No reported comorbidites	2 (5.9%)
Diabetes mellitus	7 (20.6%)
Hyperlipidemia	5 (14.7%)
Gout	1 (2.9%)
Arterial hypertension	9 (26.5%)
Previous stroke	2 (5.9%)
Cardyomiopathy	2 (5.9%)
Osteoporosis	1 (2.9%)
Psychiatric disease	4 (11.8%)
Atrial fibrillation	1 (2.9%)
Gleason score (n (%))	
6	2 (8.3%)
7a	2 (8.3%)
7b	6 (25%)
8	7 (29.2%)
9	7 (29.2%)
Previous local treatments (n (%))	
No previous local therapy	1 (4.2%)
Radical prostatectomy	20 (83.3%)
Radiotherapy	3 (12.5%)
Metastatic lesions (n (%))	
Lymph nodes only	16 (66.7%)
Bones only	4 (16.7%)
Lymph nodes and bones	3 (12.5%)
Lymph nodes, bones, visceral (liver)	1 (4.2%)

*(n)* numbe, *SD* standard deviation, *min.* minimum, *max.* maximum, *BMI* body mass index, *ECOG* Eastern Cooperative Oncology Group

Two patients were treated with [^177^Lu]Lu-PSMA-617 every 4 weeks, while 22 patients received [^177^Lu]Lu-PSMA-I&T every 6 weeks. The decision to initiate RLT, as approved by the multidisciplinary oncology committee, was influenced by various factors: in 12 patients (50% of cases), the patient’s strong preference or refusal of other treatment options; in 10 patients (41.7%), presence of significant comorbidities (e.g., heart failure, cardiomyopathy, history of stroke, or ST-elevation myocardial infarction); and for 2 patients (8.3%), no reason was documented.

### Evaluation of the response rate

The median baseline PSA level prior to [¹⁷⁷Lu]Lu-PSMA-RLT was 6.4 ng/mL (range: 0.49–3149 ng/mL). One month after completing [¹⁷⁷Lu]Lu-PSMA-RLT, the median PSA level had significantly decreased to 2.3 ng/mL (range: 0.00–1731.00 ng/mL), *p* = 0.002.

**Fig. 1:**
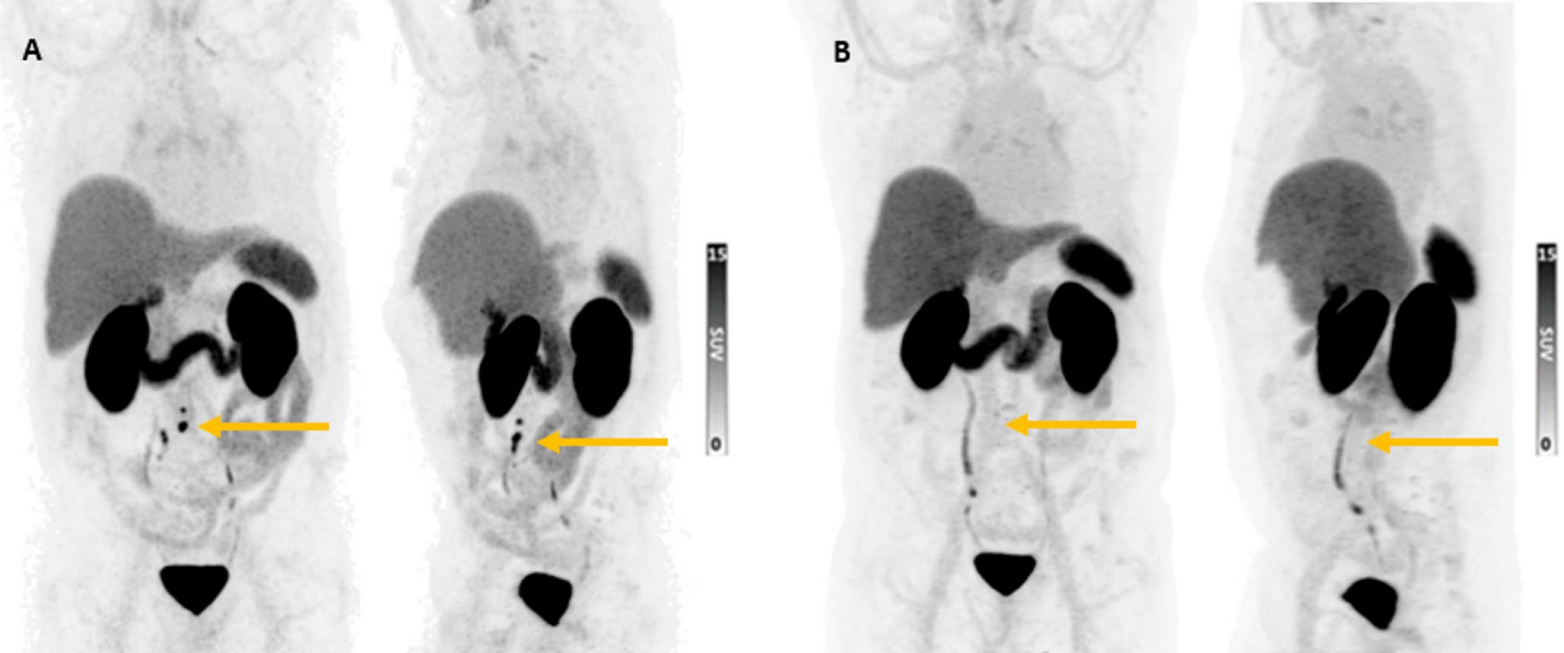
Therapy response with [^68^Ga]Ga-PSMA-11 PET images. [^68^Ga]Ga-PSMA-11 PET imaging of a patient (61 years) before (**A**) and after six RLT cycles of [¹⁷⁷Lu]Lu-PSMA-I&T (**B**). Yellows arrows in the baseline imaging indicate multiple PSMA-avid retroperitoneal lymph nodes metastases in the frontal and sagittal view. Yellow arrows in follow-up imaging show regression of PSMA-expressing lymph nodes, with no evidence of local recurrence or new osseous/visceral metastases. PSA declined from 2.54 ng/mL to 0.06 ng/mL (97.6% reduction). Over 13.9-month follow-up period, the patient showed no PSA progression and required no additional therapy

Among 24 mHSPC patients, 17 (70.8%) showed any PSA response 1 month after completing [¹⁷⁷Lu]Lu-PSMA-RLT, 15 patients (62.5% of the total cohort) achieved a ≥ 50% PSA decline, including 10 (41.7% of the total cohort) who reached a PSA decline ≥ 80% compared to their baseline PSA level. In total, 7 of these 24 patients (29.2%) showed no PSA response 4 weeks after completing [¹⁷⁷Lu]Lu-PSMA-RLT. Representative [^68^Ga]Ga-PSMA-11 PET/CT images demonstrating the therapeutic response in a patient treated with [^177^Lu]Lu-PSMA-RLT are shown in Fig. 1.

In Table 2, the volumetric and SUV-parameters derived from baseline PSMA PET-imaging are summarized. As shown, none of the baseline PSMA PET-derived metrics differed significantly between responders and non-responders.

**Table 2:** Baseline PSMA PET-derived parameters in the study cohort

Parameter	Total Cohort (*n* = 24)	Responders (*n* = 15)	Non-Responders (*n* = 9)	*P*-value
Liver SUVmean	5.3 ± 1.6			
*Total tumor volume (mL)	19.4, 5.7 (0.3–121.6.3.6)	16.8, 5.7 (0.3–121.6.3.6)	24.8, 8.3 (1.4–106.6.4.6)	0.79
Lesion SUVmax	18.4 ± 11.8	19.8 ± 13.7	17.8 ± 11.4	0.73
Lesion SUVmean	6.7 ± 3.2	6.3 ± 2.8	7.5 ± 4	0.48
Lesion SUVpeak	8.8 ± 4.9	8.9 ± 5.3	8.8 ± 4.3	0.99

*: not-normally distributed values shown in mean, median (minimum-maximum); *SUV* standardized uptake value, *max* maximum

Kaplan-Meier survival analysis demonstrated a median PSA-PFS of 24.6 months (95% CI: 16.0–33.2) in patients achieving a ≥ 50% PSA decline after completion of [^177^Lu]Lu-PSMA-RLT, compared with 5.2 months (95% CI: 0.0–10.6) in those with a PSA decline < 50% (*p* < 0.0001). In contrast, binary logistic regression analysis revealed that no baseline clinical, laboratory, or imaging parameters significantly predicted a PSA decline at 1 month after completion of [^177^Lu]Lu-PSMA-RLT.

On note, one cancer-related death occurred 10.3 months after the initiation of RLT, most likely reflecting the advanced disease stage and high total tumor volume at admission from external institution. Patient had disseminated metastases involving lymph nodes, visceral organs (liver), and axial skeleton. This 73-year-old patient received six cycles of [^177^Lu]Lu-PSMA-RLT, with PSA declining from 3149 to 1731 ng/mL (45% decline) and TTV decreasing from 685.9 ml to 523.9 ml one month after RLT. Nevertheless, in the overall cohort, median OS was not reached.

### Time to next systemic therapy

Patients with a PSA decline ≥ 50% after completion of [^177^Lu]Lu-PSMA-RLT had a significantly longer median any-therapy-free period of 27.6 months (95% CI: 22.7–32.5) compared to 8.4 months (95% CI: 4.7–12.9) in those with a PSA decline < 50% (*p* < 0.0001).

**Fig. 2:**
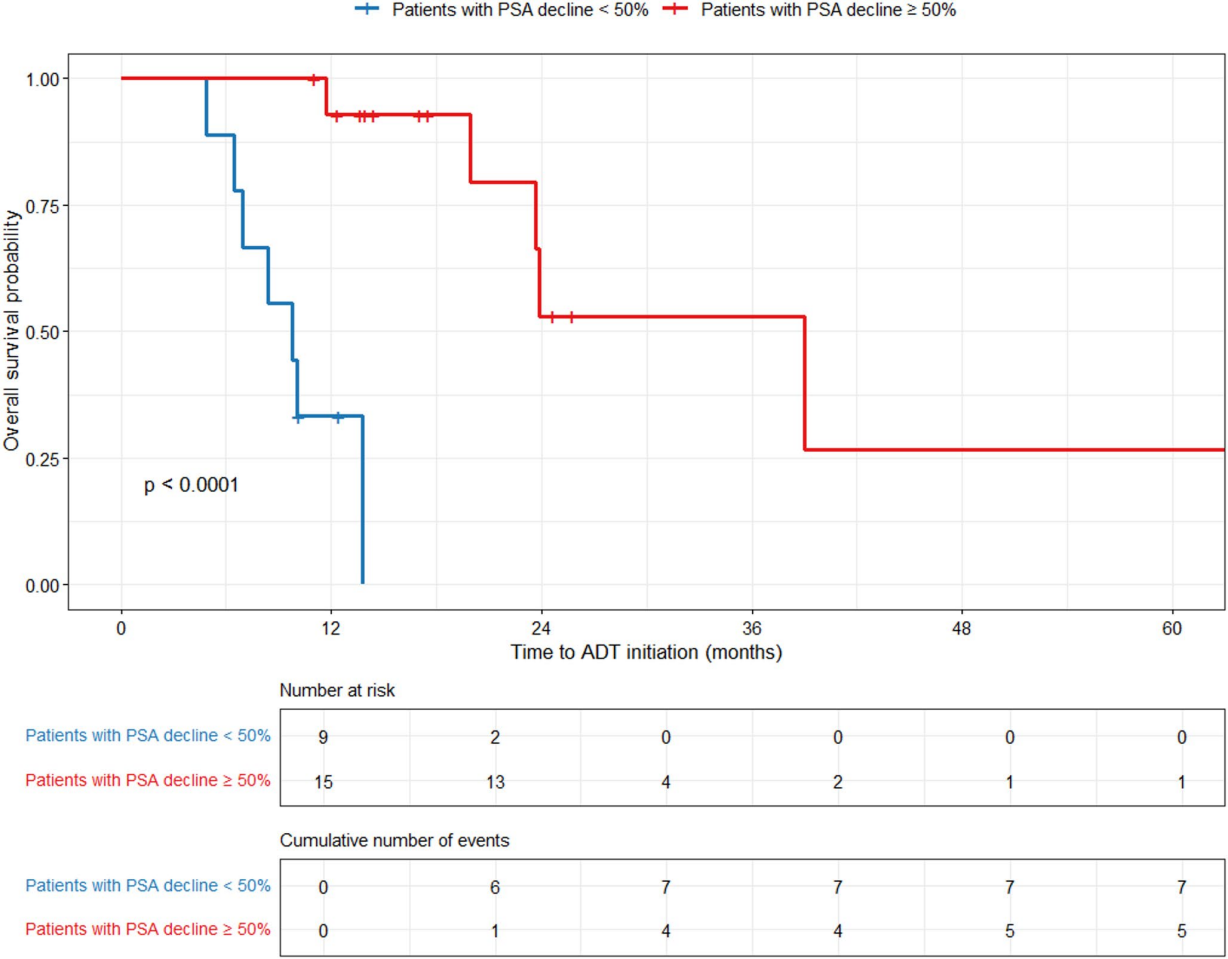
ADT-free survival stratified by PSA response after [¹⁷⁷Lu]Lu-PSMA-RLT. Kaplan-Meier curves demonstrating a significantly longer time to ADT initiation in patients with PSA decline ≥ 50% compared to those with PSA decline < 50%, with median ADT-free period of 39 months (95% CI: 23.3–54.7) vs. 9.8 months (95% CI: 5.8–13.7), respectively (*p* < 0.0001)

ADT initiation was likewise substantially delayed in responders, with a median ADT-free survival of 39 months (95% CI: 23.3–54.7) versus median ADT-free survival of 9.8 months (95% CI: 5.8–13.7) in non-responders (*p* < 0.0001), as depicted in Fig. 2.

### Predictors of ADT-free and any-therapy-free survival

Potential prognostic factors were first explored using Kaplan-Meier analysis and univariable Cox regression. In Kaplan-Meier analysis, baseline bone metastases at the initiation of [^177^Lu]Lu-PSMA-RLT were associated with shorter ADT-free and any-therapy-free survival: median ADT-free survival was 39.1 months (95% CI 18.6–59.5) in patients without bone metastases versus 10.0 months (95% CI 2.0–18.0) in those with bone involvement (*p* = 0.042), and median any-therapy-free survival of 23.4 months (95% CI 8.5–38.9) versus 9.1 months (95% CI 0–19.1.1; *p* = 0.049), respectively. Older age was also significantly associated with longer any-therapy-free survival, with a median of 23.9 months (95% CI 18.0–29.8.0.8) in patients above the median age of 66.3 years versus 11.7 months (95% CI 8.5–15; *p* = 0.043) in younger mHSPC patients. In univariable Cox analysis, higher baseline TTV was a significant determinant of shorter ADT-free survival (HR 1.02, 95% CI: 1–1.04.04, *p* = 0.03) and any-therapy-free survival (HR 1.02, 95% CI: 1–1.04.04, *p* = 0.02). No significant predictors of PSA-PFS were found.

In multivariable Cox model, baseline TTV and metastatic extent emerged independent predictors of ADT-free and any-therapy-free survival, whereas age at the initiation of [^177^Lu]Lu-PSMA-RLT did not remain significant. For any-therapy-free survival, increase in metastatic category (0 = lymphnode metastasis only, 1 = lymphnode and bone metastasis, 2 = bone metastasis only, 3 = lymphnodes, organ and bone metastasis) was associated with a 2.44-fold higher hazard of shorter any-therapy-free period (HR 2.44, 95% CI 1.03–5.79, *p* = 0.043), and higher baseline TTV was likewise associated with shorter any-therapy-free survival (HR 1.02, 95% CI: 1–1.04.04, *p* = 0.02). For ADT-free survival, a similar pattern was observed, with more extensive metastases (HR 3.1 95% CI: 1.07–9.1, *p* = 0.04) and higher baseline TTV (HR 1.02, 95% CI: 1–1.05.05, *p* = 0.02) also predicting earlier ADT initiation.

### Evaluation of [^177^Lu]Lu-PSMA-RLT toxicity

Table 3 summarizes the hematological and biochemical parameters of the patients studied before and after the last [^177^Lu]Lu-PSMA-RLT cycle. Two patients developed mild (Grade 1) anemia after completing 6 [^177^Lu]Lu-PSMA-RLT cycles and one of the patients developed mild (Grade 1) reactive thrombocytosis. Reported adverse effects included fatigue (3 patients (10.7%)), dry mouth (3 patients (10.7%)), non-specific pain (headache, pain in jaw, lumbar or pelvic region) (5 patients (17.9%)). 16 patients (57.1%) reported no treatment-related side effects. In general, all patients tolerated therapy well.

**Table 3:** Hematological and biochemical parameters before and after last [^177^Lu]Lu-PSMA-RLT cycle in all studied patients

Parameters	Prior therapy	After therapy	*P*-value
*PSA ng/mL	6.4 (0.5–3149)	2.3 (0.01–1731)	***0.002***
Hemoglobin g/dL (mean ± SD)	14.4 **±** 1.9	13.6 ± 1.6	***0.001***
*Leukocytes g/L	5.6 (4.1–13.1)	5.3 (3.5–14.2)	***0.03***
Thrombocytes g/L (mean ± SD)	236 ± 64	212 ± 47	***0.001***
Creatinine mg/dL (mean ± SD)	0.9 ± 0.2	1.05 ± 0.2	***0.001***
eGFR (mL/min/1.73 m^2^)	79.3 ± 15.8	78.2 ± 12.7	***0.001***
*Alkaline phosphatase U/L	77 (41–1567)	69 (37–316)	*NS*
*Lactate dehydrogenase U/L	171 (131–378)	167 (137–285)	*NS*
Testosterone ng/mL (mean ± SD)	3.8 ± 1.1	3.3 ± 1.4	*NS*

(*) not normally distributed data, presented in median (range) and were log_10−_ transformed for analysis; *PSA* prostate specific antigen, *SD* standard deviation, *NS* not significant, *eGFR* estimated glomerular filtration rate

## Discussion

This study represents the first retrospective analysis of systemic-treatment-naïve mHSPC patients receiving [^177^Lu]Lu-PSMA-RLT as their initial therapeutic approach.

The current standard-of-care for mHSPC has been shaped by the PEACE-1 and ARASENS clinical trials, which established triplet systemic therapy with ADT, six cycles of docetaxel, and an ARPI (abiraterone or darolutamide) as an effective treatment option. These studies demonstrated superior OS and rPFS compared to the standard doublet of ADT plus docetaxel [19, 20]. However, the backbone of systemic therapy in mHSPC is androgen suppression, which is known to pose considerable metabolic, cardiovascular, sexual, and neurocognitive risks that impair quality of life (QoL) [21–24]. In a population-based analysis, Tully et al. reported significantly higher rates of depression (12.0% vs. 7.1%) and new-onset dementia (7.4% vs. 3.4%) among ADT-treated men [25]. Similarly, Gonzalez et al. demonstrated neurocognitive decline in men initiating ADT [22]. Persistent testosterone suppression contributes to long-term sexual dysfunction with psychological and social consequences [26]. In a prospective study, Cappuccio et al. showed dramatic increase in erectile dysfunction (50% to 95.3%) and sexual inactivity (48.1% to 97.7%) after six months of ADT, with profound negative effects on self-perception [27].

In contrast, [^177^Lu]Lu-PSMA-RLT has demonstrated favorable response rates, relatively low toxicity, and less life-altering impact on QoL [11, 12]. In our cohort, most patients reported no therapy-related side effects. Mild adverse events included fatigue and dry mouth in 3 patients (10.7%) and transient pain in 5 patients (17.9%). No cases of acute nephrotoxicity during therapy or one-month post-treatment were detected in our study, despite known concerns of renal toxicity due to PSMA expression in renal tubules and radioligand excretion [28–30]. The mild decrease of eGFR (from 79.3 ± 15.8 to 78.2 ± 12.7 mL/min/1.73 m²mg/dL; *p* = 0.001) and the slight increase of creatinine (from 0.9 ± 0.2 to 1.05 ± 0.2 mg/dL; *p* = 0.001) one month after the last [^177^Lu]Lu-PSMA-RLT cycle suggest rather a possible reactive, transient change, as both parameters remained within normal range and no clinically relevant renal adverse events were documented.

The toxicity profile observed in our mHSPC cohort is similar to that reported in pretreated mCRPC patient cohort receiving [^177^Lu]Lu-PSMA-RLT in the third-line setting, where grade 1–2 anaemia and grade 1–2 xerostomia were the most frequent adverse events and grade 3–4 toxicities were observed rare [31]. Similar findings were reported in a cohort of 94 mCRPC patients with pre-existing renal impairment, in whom [^177^Lu]Lu-PSMA-RLT had only minimal impact on renal function [32]. In both treatment-naïve and pretreated patient settings, [^177^Lu]Lu-PSMA-RLT predominantly induces myelotoxicity with potential short-term effects on PSMA-expressing normal tissues (salivary glands, kidneys), therefore toxicity primarily being determined by radioligand biodistribution and organ dosimetry. In our cohort, most patients reported no therapy-related side effects. Mild adverse events included fatigue and dry mouth in 3 patients (10.7%) and transient pain in 5 patients (17.9%). Given the low number of patient-reported symptoms, we hypothesize that, owing to less extensive prior therapy, less comorbid, treatment-naïve patients retain better baseline organ functions, which may offer greater resilience and support the favorable tolerability profile observed in our mHSPC cohort. However, larger retrospective analyses and prospective studies are warranted to validate this hypothesis.

Efficacy outcomes in our study were encouraging. Patients achieving a PSA decline ≥ 50% (62.5%) experienced a median PSA-PFS of 24.6 months, any-therapy-free survival of 27.6 months (95% CI: 22.7–32.5), and ADT-free survival of 39.0 months (95% CI: 23.3–54.7). By contrast, non-responders had markedly shorter PSA-PFS (5.2 months), any-therapy-free survival (8.4 months, 95% CI: 4.7–12.9), and ADT-free survival (9.8 months, 95% CI: 5.8–13.7). These favorable outcomes might reflect the typically lower tumor burden, as patients with lower baseline TTV exhibited a significantly longer ADT-free and any-therapy-free survival; this association, alongside less extensive metastatic involvement, remained significant in both univariable and multivariable analyses. We consider it important to distinguish between time to ADT initiation and time to any-therapy, since ADT is associated with more pronounced, life-altering side-effects, especially in younger patients.

Further identification and evaluation of baseline clinical indicators to refine selection of mHSPC patients most likely to benefit from [^177^Lu]Lu-PSMA-RLT RLT is highly required. In this cohort, only baseline TTV and metastatic distribution emerged as significant predictors of ADT-free and any-therapy-free survival. However, baseline SUV-parameters (SUVmax, SUVmean, SUVpeak) did not differ significantly between responders and non-responders. Consequently, our data do not support the hypothesis that higher SUV values reflect increased PSMA expression and are associated with greater [¹⁷⁷Lu]Lu-PSMA-RLT uptake and improved therapeutic outcomes [33]. This finding may, in part, be attributable to the absence of prior systemic therapies in this patient cohort, potentially resulting in more homogeneous PSMA expression and, therefore, less variability in SUV parameters. Nevertheless, the biological determinants underlying heterogeneity in PSMA expression remain insufficiently characterized. Hormone-deprivation therapies can remodel tumor biology by creating an immunosuppressive microenvironment, altering PSMA expression, and modifying radiation sensitivity [34, 35]. Similarly, taxane-based chemotherapy can downregulate PSMA expression and compromise organ function, potentially reducing the efficacy and tolerability of subsequent therapies [36]. By contrast, [^177^Lu]Lu-PSMA-RLT does not appear to alter tumor biology, enabling early, uniform targeting of PSMA-expressing disease.

Our findings align with ongoing efforts to evaluate [^177^Lu]Lu-PSMA-RLT in earlier treatment lines. The completed phase II UpFrontPSMA trial demonstrated superior PSA responses, PSA-PFS, and CRPC-free survival with sequential [^177^Lu]Lu-PSMA-617 plus docetaxel versus docetaxel alone [11]. Active trials, including the phase III PSMAddition (NCT04720157) and the phase I/II LuTectomy (NCT04430192), are investigating RLT in combination with ADT and ARPI or in the neoadjuvant setting before radical prostatectomy, and their results are highly anticipated [12, 37].

Nonetheless, this study has several limitations. The retrospective design, relatively small cohort size, and potential documentation or follow-up bias across a 10-year treatment period must be acknowledged. Heterogeneity in treatment protocols is another limitation: patients received [^177^Lu]Lu-PSMA-617 every 4 weeks prior to 2021 and [^177^Lu]Lu-PSMA-I&T every 6 weeks thereafter. Additionally, use of two different compounds represent a potential concern; though, prior data by Schuchardt et al. indicate comparable therapeutic effect, safety and biodistribution profiles for these compounds, without significant hematologic or nephrotoxic differences [38].

## Conclusion

First-line treatment with [^177^Lu]Lu-PSMA-RLT demonstrated a favorable safety profile, effective disease control with prolonged PSA-PFS, any-therapy-free and ADT-free survivals in treatment-naïve mHSPC patients. Obtained results warrant further investigations of [^177^Lu]Lu-PSMA-RLT as an initial therapy for patients with mHSPC.

## Data Availability

The datasets analyzed during the current study are available from the corresponding author on reasonable request.
